# Arsenic and heavy metal contamination in drinking water from an industrial zone in Dhaka District, Bangladesh

**DOI:** 10.1371/journal.pone.0332601

**Published:** 2025-10-16

**Authors:** Md Kamal Hossain, Salma Sultana, Afroza Parvin, Fahima Islam, Mohammad Moniruzzaman, Badhan Saha, Afsana Parvin, H. M. Solayman, Sumaia Sharmin

**Affiliations:** 1 BCSIR Laboratories Dhaka, Bangladesh Council of Scientific and Industrial Research (BCSIR), Dhaka, Bangladesh; 2 Cental Analytical Research Facilities (CARF), Bangladesh Council of Scientific and Industrial, Dhaka, Bangladesh; 3 Department of Fisheries and Marine Science, Noakhali Science and Technology University, Noakhali, Bangladesh; 4 Faculty of Civil Engineering Technology, Universiti Malaysia Pahang Al-Sultan Abdullah, Gambang, Pahang, Malaysia; 5 Department of Soil, Water and Environment, Dhaka University, Dhaka, Bangladesh; UTRGV: The University of Texas Rio Grande Valley, UNITED STATES OF AMERICA

## Abstract

Heavy metals (HMs), even in trace concentrations, can pose serious health risks when consumed over time. In Bangladesh, the widespread use of tube wells for drinking water, coupled with industrial activity, has contributed to the contamination of groundwater with HMs. This study investigated heavy metal contamination in drinking water samples from Gazipur, an industrial hub, using Inductively Coupled Plasma-mass Spectrometry (ICP-MS). The results revealed that the mean concentrations of HMs in mg/L followed the order: Fe (5.479 ± 3.740)> Mn (0.203 ± 0.233)> Pb (0.133 ± 0.370)> Zn (0.068 ± 0.070)> Cu (0.016 ± 0.034)> As (0.003 ± 0.004)> Ni (0.002 ± 0.001)> Cr (0.002 ± 0.001). Concentrations of Pb, Fe, and Mn exceeded safe limits while As, Cd, Cr, Ni, Cu, and Zn were within acceptable ranges. The scatter plot analysis revealed weak and non-significant correlations between As concentrations and other heavy metals with low *R*² values. A strong difference in metal contamination levels between shallow (20–80 meters) and deep wells (>80 meters), with shallow wells exhibiting significantly higher contamination percentages, often approaching 100%, while deep wells consistently remained below 30%. Ecological risk assessments showed low to moderate contamination at most sampling sites. Health risk evaluations (HQ and HI) indicated that all metals remained below harmful levels, though arsenic posed a heightened cancer risk, particularly for children. Principal Component Analysis (PCA) and Cluster Analysis suggested that As, Pb, Fe, Zn, and Mn were linked to industrial activities, while the other metals were likely of geological origin. The study emphasized the need for ongoing surveillance and intervention to protect public health in areas impacted by industrial pollution.

## Introduction

Water, an indispensable and finite resource, stands as the cornerstone of life’s existence on our planet, irreplaceable in its essence [1]. Safe drinking water is essential for human survival, and should not offer any substantial risks [2]. In Bangladesh, underground water from handheld tube wells is the main supplier of drinkable water [3–5]. However, groundwater contamination in Bangladesh is considered to be the most serious issue. Among different groundwater contaminations, iron and arsenic are the most harmful, which are present in trace amounts but have significant effects on drinking water and cause harmful impacts on human health in developing countries of Southeast Asia [6]. Although ingesting some metals, such as As, Fe, Pb, and Cd, is highly hazardous for human health even at a low concentration, some metals, such as Cu, Ni, Zn, and Mn, are helpful to humans at low quantities [7]. Extended exposure to these metals can lead to various harmful health effects, including an increased risk of cancer. Different diseases in humans, including carcinoma, melanosis, dermatitis, and black foot syndrome can be caused by the As and other heavy metals [8,9]. Both natural processes and human activity discharge HMs into the environment [10–12]. Mineral and heavy metal concentrations in environmental matrices such as soil [13], sediment [14], water, air [15], or biological tissues [9,14] vary widely due to a complex interplay of natural and anthropogenic factors. An unusual feature of groundwater pollution by HMs from tube wells is “reductive dissolution” [16]. Certain geological and hydrological conditions are necessary for this process to take place, especially in areas where the amount of organic matter in the groundwater is enormous and the level of oxygen is low. In reductive dissolution, certain heavy metal compounds, such as iron and manganese oxides, which naturally occur in the aquifer sediments, act as carriers for other heavy metals like arsenic, lead, and cadmium [17]. When groundwater loses oxygen due to microbial activity or other variables, the metal oxides undergo reduction processes, releasing the bound HMs into the water [16,18].

The burgeoning expansion of manufacturing and commercial enterprises in emerging economies like Bangladesh has stirred profound apprehensions over water reserves in recent times [17]. The groundwater contamination is attributed to hazardous anthropogenic activities on the surface of the earth [19]. Industrial areas serve as the epicenter of these activities. The groundwater pollution becomes worse when it is enriched with heavy metals [20–22]. Various industrial activities enrich water with heavy metal concentrations of varying levels [23]. Moreover, the escalation of As contamination in potable groundwater within this nation has exacerbated the risk of arsenic poisoning. In Bangladesh, the accepted peak levels of arsenic in drinking water stand at around 0.05 mg/L, as documented by Ref [24] and Ref [25]. Ref [26] found a significant disparity in As concentrations across the country, ranging from 1 to 224 ppb at depths between 23 and 45 meters which highlighted significant variations in As levels in groundwater across different regions and depths throughout Bangladesh. The measured levels of arsenic in ground water in 50 districts of Bangladesh exceeded the stated limit of 50 μg/L [27,28]. Small rural communities and individuals frequently partake of water containing elevated levels of HMs surpassing recommended thresholds. Tube wells in the tableland and hillside tracts had no contamination of As, but the floodplain and deltaic regions, including the seashore region, were extremely polluted in Bangladesh [29,30]. As a megacity and economic hub, Dhaka has grown chaotically to absorb mammoth permanent and temporary inhabitants that need more attention from the scientific community. Contamination with hazardous metallic elements poses a pervasive threat to water safety in both community and private sources throughout Bangladesh, with rural and urban regions at greater risk of violating safe drinking water standards for these and other contaminants [31]. Despite the focus on surface water in other major cities, there is a lack of notable studies addressing groundwater quality, particularly in relation to extensively utilized tube well sources in Bangladesh.

Regulatory bodies propose maximum allowed levels for heavy metals in drinking water to mitigate their impacts. Therefore, assessing the risk of exposure for human populations involves evaluating factors such as contamination degree index (C_deg_), hazard quotient (HQ), ecological risk index (ERI), and carcinogenic risk (CR) [32]. Monitoring and managing these indices are essential for protecting human health, sustaining ecosystems, and ensuring the availability of clean and safe water resources for current and future generations [33]. In this study, the emphasis is on the significance of preserving the safety of drinking water. The objectives were (i) to ascertain the existing levels of heavy metals in Tubewell water in Gazipur as well as its physicochemical characteristics; (ii) to illustrate the degree of pollution in water using a variety of indices, including the Degree of Contamination Index (Cdeg), the Heavy Metal Pollution Index (HPI), and the Heavy Metal Evaluation Index (HEI), (iii) to evaluate the ecological risk index (ERI) and human health risk assessment (HRA) associated with metals and (iv) to pinpoint potential sources of heavy metal contamination in the drinking water samples collected from a notable industrial and economic center in Bangladesh through PCA and HCA.

## Methods

### Study area

**Fig 1:**
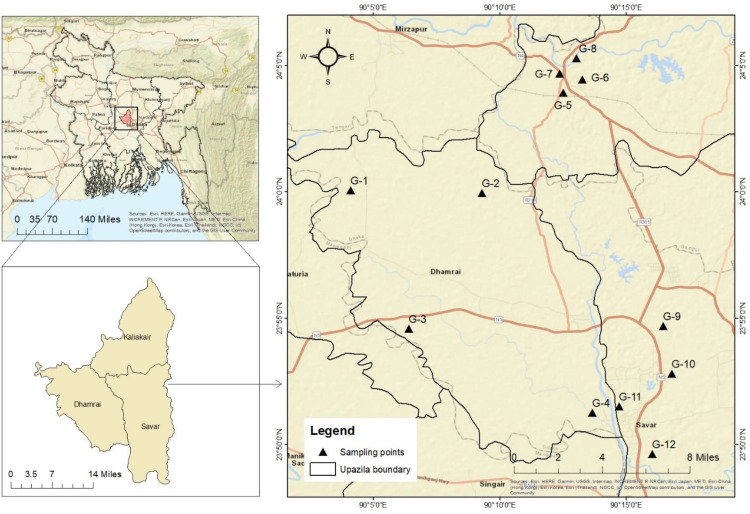
Various sampling location point of the respective selected industrial area in an around of Dhaka district (Source: ArcGIS Desktop 10.8.2, including ArcMap).

The study sites were carefully chosen within Gazipur district’s primary industrial region, which includes three distinct areas: Dhamrai, Kaliakair and Savar (Fig 1). This region is renowned for its industrial activities, spanning textiles, garments, pharmaceuticals, food and beverage processing. However, it’s also known to be vulnerable to arsenic contamination in groundwater, as highlighted by Ref [30].

The industrial operations in Gazipur have unfortunately left a significant mark on the environment. The discharge of waste from their operations, products, and raw materials has led to alarming levels of pollution in and around Gazipur district. This pollution poses a serious threat to both environmental sustainability and public health in the region.

### Water sample collection and preparation

A total of 60 drinking water samples were collected randomly from various hand tube-wells and electrically driven pumps across the study area, with depths ranging from 20 to 100 meters. Polyethylene terephthalate bottles were utilized for this purpose, ensuring the integrity of the samples. Prior to collection, each bottle underwent meticulous washing and rinsing with water from the respective tube-well or electric pump. Additionally, to minimize contamination and ensure the collection of genuine aquifer water, each hand tube-well or electric pump was purged for approximately 5 minutes before sampling, thereby preventing the inclusion of extraneous floating solids from the pipes. The water sample was taken in 1000 mL PET bottles washed with 2% HNO_3_. The PET bottles underwent three rinses with tube well water prior to sampling. Three water samples from each sampling station were taken for physiochemical and heavy metal analysis. The pH, EC, DO and TDS were measured on-site by using calibrated Hach multi parameters during the sampling period (SenIon156, HACH, USA). A portion of each sample was separated for metal analysis and acidified with 2–3 drops of analytical reagent-grade concentrated HNO_3_ to attain a pH level of less than 2 [34]. The bottles were sealed, labeled, brought to the lab, and refrigerated until analysis.

To ensure the integrity of the samples, all bottles containing the sampled water were securely sealed with screw caps and stored in a refrigerator at 4°C for further analysis. This meticulous preservation method, combined with efforts to minimize sample handling, was employed to maintain the samples’ stability and integrity for subsequent laboratory examinations.

### Heavy metal analysis by ICPMS

In the research facility, heavy metal concentrations (As, Pb, Cd, Cr, Cu, Ni, Zn, Mn, Fe) were determined using Inductively Coupled Plasma Mass Spectrometry (ICP-MS) (PerkinElmer, Model: NexION 2000; USA). In a spectroscopic elemental analysis sample preparation, acid digestion is an important step of the entire analytical procedure [35,36]. For metal analysis, 100 mL of acid-preserved water sample was digested with concentrated HNO_3_ [36]. After cooling to room temperature, it was then diluted to 100 mL with deionized water and then filtered through Whatman No. 42 filter paper. After digestion, samples were refrigerated at 4^◦^C for chemical analysis.

### Ethical statement

All of the samples collected were usually consumed by the local community. As a result, no permits were required for the conduct of this investigation. Furthermore, there were no ethical issues about the experimental techniques, as there were no harmful or invasive methods used. All sampling was carried out responsibly, with minimal disruption to the environment and human well-being. No experimental manipulations or interventions were performed.

### Inclusivity in global research

Additional information regarding the ethical, cultural, and scientific considerations specific to in inclusivity in global research is included checklist.

### Quality control and quality assurance

Quality control (QC) and quality assurance (QA) are fundamental components of laboratory operations, ensuring that the data produced is of the highest quality and meets the needs of stakeholders while adhering to regulatory requirements. To lessen the chance of contamination during sample preparation and analytical procedures, all glassware used in this experiment was cleaned and rinsed with ultrapure acid and water. Prior to standard calibration, NexION Setup solution (PerkinElmer, USA) evaluated the ICP-MS equipment’s performance. Over a dynamic range of 1.0 to 100 ppb, a five-point calibration variance, R^2^ > 0.9995, was attained. For the purpose of verifying instrument stability, at least three replicates were performed. A maximum relative standard deviation (RSD) of 5–8% was taken into consideration. When the response for a particular metal was three times and ten times larger than the background noise, the instrument’s detection limit (DL) and quantification limits (QL) were established. To determine the recovery % of the elements (As, Se, Cr, Cu, Co, Pb, Ni, Zn, Hg, V, Fe, and Mn) in the sample, a standard reference materials SRM NIST 1643f from the National Institute of Standards (NIST) was utilized. Using NIST 1643F, the proportion of significant elements recovered from the sample varied between 95.97 and 104.21% (S1 Table). Table 1 represents the Limit of Detection (LOD) and Limit of Quantification (LOQ) for the element analysis in ICPMS. To reduce error, the quality control procedure included the recovery of SRM samples, spike samples, filter blanks, and reagent blanks. Based on the recovery and examination of blank samples, adjustments were made to the trace metal concentrations.

**Table 1:** Limits of Detection (LOD) and quantification (LOQ) for elements analysis in ICP-MS.

Elements	Unit	LOD	LOQ
As	ppb	0.012	0.12
Se	ppb	0.01	0.1
Pb	ppb	0.03	0.3
Cd	ppb	0.005	0.05
Co	ppb	0.01	0.1
Cr	ppb	0.05	0.5
Cu	ppb	0.05	0.5
Ni	ppb	0.05	0.5
Zn	ppb	0.05	0.5
Fe	ppb	0.11	1.1

### Risk assessment in studied water samples

The risk assessment of water samples for heavy metal analysis involves evaluating the potential health and environmental risks associated with exposure to heavy metals in water sources. This process helps identify and quantify the likelihood and magnitude of adverse effects on human health and ecosystems resulting from heavy metal contamination. The ecological risk indices—Heavy Metal Pollution Index (HPI), Heavy Metal Evaluation Index (HEI), Degree of Contamination Index (C_deg_) and Ecological Risk Index (ERI)—as well as the human risk indices—Hazard Quotient (HQ), Hazard Index (HI) and Carcinogenic Risk (CR)—were used in the present study to assess the risk of heavy metals in drinking water samples, utilizing equations (Eqs.) (1), (2 a, b), (3 a, b, c), (4 a, b) and (5a, b). The mean concentrations of the metals under analysis served as the basis for computing the HPI values [37]. Integral to this assessment are considerations of various factors including the dosage and duration of exposure, as well as individual susceptibility predicated upon genetic predisposition, lifestyle choices, and other pertinent variables [38]. A detailed description of these indices is provided (S2 Table).


$$HEI=\sum_{i=\text{1}}^{n}Mi/MACi\;$$ (1)

$$C_{d}=\sum {\mathrm{\backslash nolimits}}_{i=\text{1}}^{n}Cfi$$ (2a)

$$Cfi=\frac{Mi}{MACi}-\text{1}$$ (2b)

$$E_{r}^{i}=T_{r}^{i}\times CF$$ (3a)

$$CF=\frac{C_{metal}}{C_{background}}$$ (3b)

$$ERI=\sum E_{r}^{i}$$ (3c)

$$HQ=\frac{CDD}{RfD}$$ (4a)

$$HI={HQ}_{\text{Ingestion}}+{HQ}_{\text{Inhalation}}+{HQ}_{\text{Dermal}}$$ (4b)

CR=CDD×SF (5a)

TCR=∑CR (5b)

### Statistical analysis

The study utilized multivariate statistical techniques as a quantitative and independent approach to streamline, condense, and comprehend variations in order to extract meaningful insights, such as modeling and interpreting large datasets [11,12]. Linear Regression, Principal Component Analysis (PCA) and Cluster analysis were conducted using SPSS software (Version 20), PAST (4.0), and other graphs were tailored by MS excel and OriginPro (8.0). A 95% confidence interval was applied during the implementation of these statistical methods (with significance set at p < 0.05).

## Results and discussion

### Drinking water in Gazipur region: features and water quality status

Physical, chemical, and biological factors can be used to determine the quality of water in any given area or source [39]. Table 2 presents a comprehensive overview of the physicochemical parameters, namely pH, Total Dissolved Solids (TDS) and Electrical Conductivity (EC), ascertained from a series of drinking water samples procured from diverse locations within Gazipur. The pH scale, ranging from 0 to 14, serves as a measure of the hydrogen ion concentration where values below 7 indicate acidity, 7 denotes neutrality and values above 7 signify alkalinity [44]. In the present study, pH levels ranged slightly alkaline, varying between 6.73 and 8.11 with an average of 7.48. The EC values indicated a moderate range, spanning from 334 to 634 μS/cm, with an average of 464.75 μS/cm. These readings can fluctuate due to factors like dissolved minerals and ions present in the water. Higher conductivity typically suggests a higher concentration of dissolved solids which may include minerals such as calcium, magnesium and sodium along with other ions. It is important to note that a TDS concentration exceeding 1000 μg/L is deemed intolerable for human consumption while levels surpassing 2000 μg/L are considered unsafe for plants and crops [45]. The TDS concentrations in the samples varied considerably, ranging from 165.1 to 316 mg/L with an average of 220.33 mg/L. Notably, the pH, EC and TDS concentrations in the water samples fell within the drinking water guidelines established by the Ref [5].

**Table 2:** Parameters of drinking water in Gazipur region.

Parameter	Unit	Range	Mean ± SD	Drinking water standard	
				[40,41]	[42,43]
pH	–	6.73 - 8.11	7.42 ± 0.29	6.5 - 8.5	6.5 - 8.5
TDS	mg/L	165.1 - 316	220.33 ± 44.74	1000	1000
EC	µS/cm	334 - 634	464.34 ± 88.13	400	–

### Heavy metal concentrations in studied water samples

The heavy metals exhibited varying concentrations (mg/L), with Fe being the highest, followed by Mn, Pb, Zn, Cu, As, Ni, and Cr, as depicted (Table 3 and S3 Table). The mean concentration of Fe in the drinking water samples was 5.479 ± 3.740 mg/L and ranged between 0.152 mg/L and 10.842 mg/L. The average value of Fe was found extremely higher than the standard guideline value of the Ref [51] and Ref [52]. Additionally, the mean concentration of Fe was significantly greater to the previously reported value of 3.593 mg/L in Satkhira [47], 3.235 mg/L in Laksmipur [48], 0.3452 in Dinajpur [49] and 0.0033 mg/L in Noakhali [50]. High levels of iron in drinking water can lead to a metallic taste, staining of plumbing fixtures, and discoloration of water. While iron itself is not typically harmful to human health in small amounts, elevated levels may indicate other contaminants or issues with water treatment [53].

**Table 3:** Heavy metal concentration Mean± SD (mg/L) in studied drinking water samples (TW) and comparison with similar studies.

Locations	Heavy metal concentration (mg/L)									
	As	Pb	Cd	Cr	Cu	Ni	Zn	Mn	Fe	References
Gazipur	0.003 ± 0.004	0.133 ± 0.370	0.002 ± 0.006	0.002 ± 0.001	0.016 ± 0.034	0.002 ± 0.001	0.068 ± 0.070	0.203 ± 0.233	5.479 ± 3.740	Present Study
Gazipur	0.89	0.037	0.013	0.0074	0.05	0.0211	0.33	0.1249	NA	[46]
Satkhira	0.057	NA	< 0.001	NA	NA	NA	0.038	0.143	3.593	[47]
Lakshimpur	0.085	0.00037	NA	NA	NA	0.00191	0.022	0.652	3.235	[48]
Dinajpur	NA	0.0234	NA	0.0106	0.0375	NA	0.0156	0.4069	0.3452	[49]
Noakhali	0.2975	NA	NA	NA	0.0045	NA	0.0374	0.1396	0.0033	[50]
Maximum admissible limit	0.01	0.01	0.003	0.05	2	0.02	3	0.1	2	[51]
	0.05	0.05	0.005	0.05	1.0	0.07	5.0	0.05	NA	[5]
	0.05	0.05	0.005	0.05	1.0	0.1	5.0	0.1	0.3 - 1.0	[52]

(NA: Not available).

The Mn content ranged between 0.02 − 0.926 mg/L with an average concentration of 0.203 ± 0.233 mg/L which exceeded the standard guideline value set by the WHO established in both 2004 and 2011 [5,51]. This elevation may stem from both natural geochemical processes and anthropogenic influences. Geologically, Mn is commonly found in sedimentary rocks and soils, and under anaerobic or reducing conditions—which are typical in deep tubewell aquifers—Mn can become more soluble and mobilized into groundwater. In addition, industrial discharge, particularly from textile dyeing, metal processing, or battery recycling facilities—all of which are present in and around Gazipur—may contribute to Mn contamination through direct or indirect release into groundwater. Agricultural runoff containing Mn-based fertilizers or fungicides could also be a contributing factor. However, the mean value, although lower than the reported Mn concentrations in Laksmipur (0.652 mg/L) [48] and Dinajpur (0.4069 mg/L) [49], surpassed those in Gazipur (0.1249 mg/L) [46], Satkhira (0.143 mg/L) [47], and Noakhali (0.1396 mg/L) [50], as illustrated in Table 3. In low concentrations, Mn contributes to water’s taste and odor, similar to Fe, and is generally considered harmless. However, elevated levels of Mn in drinking water can pose health risks. From a health perspective, while Mn is an essential trace element, chronic exposure to elevated levels through drinking water has been linked to neurological effects, particularly in children, including cognitive and behavioral impairments. Prolonged high intake may also pose risks to the liver and central nervous system.

The mean concentration of Pb in the drinking water samples was measured at 0.133 ± 0.370 mg/L, with a range spanning from 0.007 mg/L to 1.358 mg/L. This average value exceeded the standard guideline values established by both the WHO and the ECR [5,51,52]. Furthermore, the mean concentration of Pb surpassed reported levels in other regions, notably Gazipur (0.037 mg/L), Laksmipur (0.00037 mg/L), and Dinajpur (0.0234 mg/L). Exposure to elevated levels of Pb in drinking water can have serious health implications, particularly for vulnerable populations such as children and pregnant women [11].

The average concentrations of As, Cd, Cr, Ni, Cu and Zn were recorded at 0.003 ± 0.004, 0.002 ± 0.006, 0.002 ± 0.001, 0.002 ± 0.001, 0.016 ± 0.034 and 0.068 ± 0.070 mg/L, respectively, falling below the standard guideline values established by the Ref [5]. These values were also lower than the reported concentrations in drinking waters from different locations of Bangladesh. The analysis of one-way ANOVA revealed a statistically significant difference in metal concentrations with an F-value of 27.99 and a p-value of 3.00e-22 (p < 0.05), indicating that not all metals exhibit the same concentration levels.

**Fig 2:**
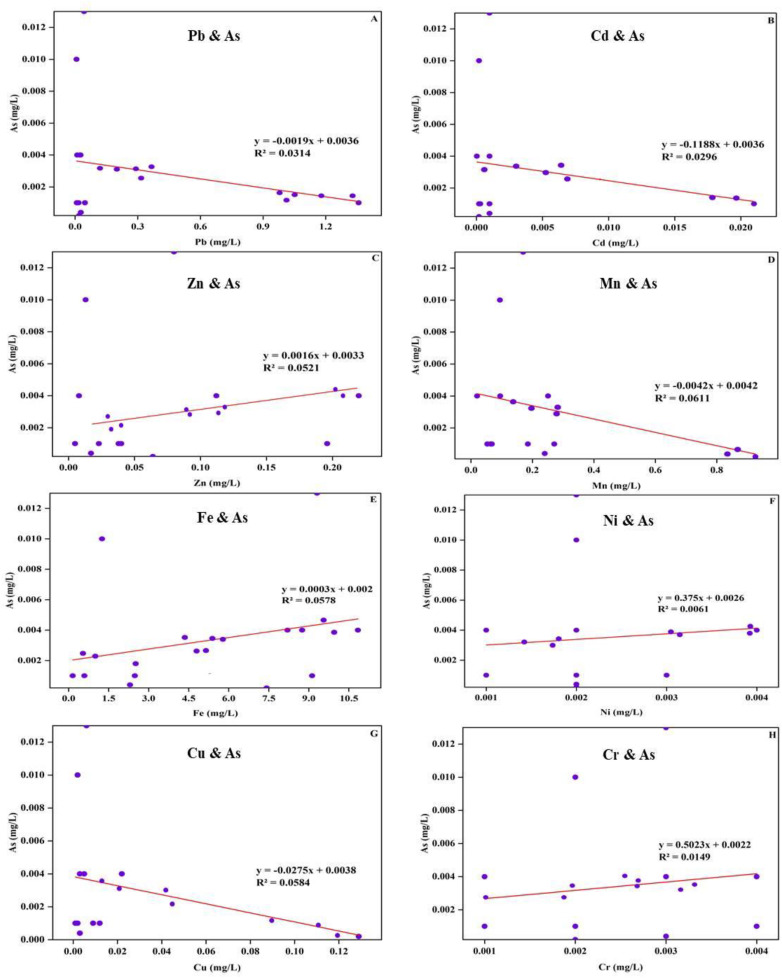
Linear regression showing the relationships between contamination of As and other studied metals of the selected area.

In this research, particular emphasis was placed on As contamination as it is a key component of over 200 minerals, including elemental arsenic, arsenides, sulfides, oxides, arsenates and arsenites, as noted by the Ref [54]. In their comprehensive study “Arsenic Contamination of Groundwater in Bangladesh,” Ref [54] illustrated that the highest concentrations of these minerals are found in mineralized regions, often closely associated with transition metals such as cadmium (Cd), lead (Pb), silver (Ag), gold (Au), antimony (Sb), phosphorus (P), tungsten (W) and molybdenum (Mo). The majority of widespread As ore mineral is arsenopyrite, or FeAsS. Chemical data for ground waters from deep tube wells of Dhaka city (S4 Table) revealed that in Gazipur region, the concentrations of several heavy metals were notably higher compared to other locations reported by the Ref [54]. Specifically, Pb (0.133 mg/L) in Gazipur is significantly elevated compared to the much lower values in other areas (0.00017 to 0.00037 mg/L), indicating considerable Pb contamination. Similarly, Cd (0.002 mg/L) in Gazipur surpassed the trace amounts found in other locations where Cd is consistently below 0.001 mg/L. Cu (0.016 mg/L) was also higher than in the comparison locations where Cu levels do not exceed 0.002 mg/L. The concentration of Mn (0.203 mg/L) in Gazipur is considerably elevated compared to the other sites (0.017 to 0.066 mg/L). Most strikingly, iron (5.479 mg/L) in Gazipur was drastically higher than in other regions where Fe concentrations range from 0.021 to 0.248 mg/L. The “iron oxide reduction hypothesis” proposes that As is desorbed and dissolved by iron oxides, which had previously scavenged arsenic from river water during the transportation of sediments. Alteration in iron oxide levels during sediment deposition may influence the distribution of elevated arsenic levels in groundwater. Insufficient evidence indicates that As hotspots in the north of Bangladesh may be associated with iron oxide-rich sediments that contain adsorbed arsenic. On the other hand, the geographic distribution of As and Mn concern areas differed dramatically, with only 33% of shallow well fluids meeting WHO guidelines for both. However, throughout the whole country, concentrations of As spanned almost four orders of magnitude, from <0.0005 mg/L to in excess of 2.3 mg/L. According to DPHE/ BGS National Hydrochemical Survey, the mean concentration of As in Gazipur district was 0.004 mg/L [54]. The Figs 2A-2H contains eight scatter plots with corresponding regression equations and *R*^2^ values that explore the relationships between As concentrations and other studied heavy metals. The analysis revealed weak correlations in all cases. Pb, Cd, Mn, and Cu exhibited negative correlations with As, with *R*^2^ values of 0.0314, 0.0296, 0.0611, and 0.0854, respectively, indicating minimal explanatory power for these relationships. In contrast, Zn, Fe, Ni, and Cr showed positive correlations, but the *R*^2^ values (ranging from 0.0061 to 0.0578) remained similarly low. These findings suggest that none of the heavy metals individually explain a significant amount of variance in arsenic concentrations, highlighting the weak and non-significant relationships between As and these metals in the studied samples. There are many reasons behind the observed correlations. Firstly, difference in origin can lead to varied distribution patterns and thus weak correlations which is elaborated in Pollution source identification of studied metals section. Secondly, although Fe and As can be concurrently released through reductive dissolution, the reprecipitation of dissolved iron as siderite (FeCO_3_) under reducing conditions leads to a poor correlation between Fe and As in groundwater. Thirdly, arsenic’s mobilization is driven by complex and independent pathways beyond just co-occurrence with other heavy metals [17]. Additionally, dissolved organic matter (DOM), particularly its small molecular weight and protein-like components, demonstrates strong positive correlations directly with arsenic concentrations, acting as a significant independent factor for As mobilization through complexation or desorption [4,17].

**Fig 3:**
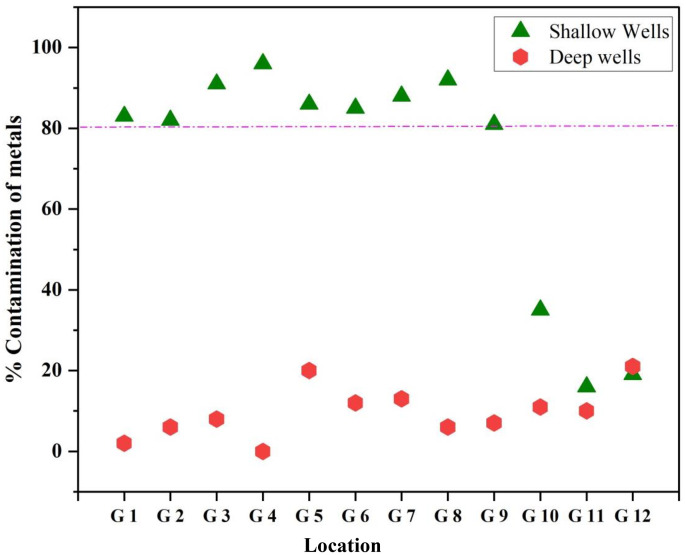
Percentage of wells contaminated of metals in the selected area of industrial zone in an around Dhaka district.

As is one of the most useful chemical constituents for distinguishing between deep and shallow groundwater supplies. Deep well groundwater typically has low levels of As even in extensively contaminated locations. Although the original deep well data had only covered a small portion of Bangladesh, further drilling in other areas except for the Chittagong Hill Tracts, 3534 wells were sampled throughout Bangladesh, and the findings revealed that of all shallow wells (depths under 150 meters), 27% were polluted. Having arsenic (As) levels higher than those in Bangladesh (0.05 mg/L) including West Bengal, supported this finding. Out of all wells with a depth greater than 150 meters, just 1% surpassed the Bangladeshi norm and 5% exceeded the WHO guideline value. Arsenic concentrations in Bangladesh exhibit an extensive range, varying from below 0.00025 mg/L to over 2.0 mg/L. Water samples were collected from seven deep wells in Dhaka city, all of which draw water from the old Dupi Tila aquifer in the Madhupur Tract. The arsenic levels in these wells were extremely low, with all measurements falling below the detection limit of 0.00025 mg/L [54]. In case of our recent investigation, we observed, a strong difference in metal contamination levels between shallow (20–80 meters) and deep wells (> 80 meters) across the locations studied (Fig 3). For shallow wells, the metal contamination percentage was generally much higher than for deep wells, with several values approaching or reaching 100%, particularly in the location G3 to G9 range. Furthermore, deep wells showed significantly lower contamination percentages, staying below 30% in all cases. The consistently low levels in deep wells across all groups suggested that deeper groundwater sources might be less exposed to metal contamination, possibly due to natural filtration or the lower influence of surface contaminants. There are also other factors that can explain the variability. For example, the aquifers in Bangladesh exhibit a high degree of sedimentological complexity and spatial variability, both laterally and with depth. Moreover, the aquifers are highly stratified. This stratification means that different layers can have distinct geochemical conditions and metal loads. Vertical mixing and the connectivity between shallow and deep aquifers are also critical for understanding the potential spread of contamination [54]. Additionally, both shallow and deep tubewells yielded iron-rich groundwater, a finding consistent with the observations of the Ref [54].

### Evaluation of ecological risk indices in water samples

**Fig 4:**
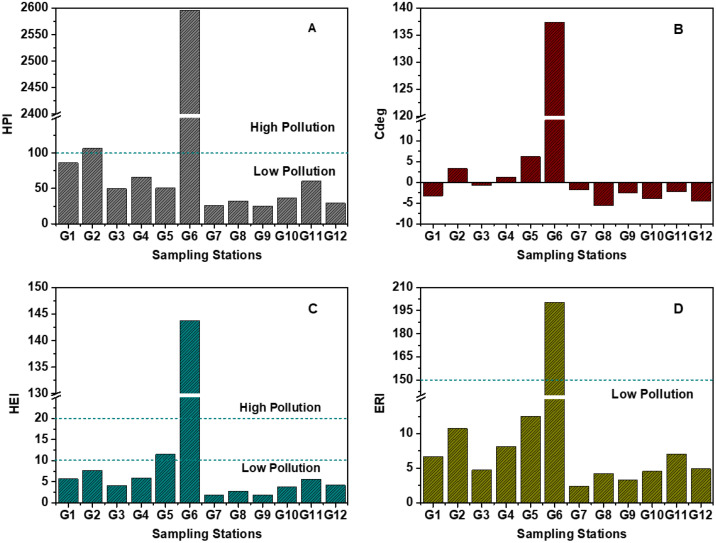
Heavy metal pollution index (HPI), degree of contamination index (Cdeg), heavy metal evaluation index (HEI), and ecological risk index (ERI) of metals in tube well water of the selected area.

The mean concentrations of the metals under analysis served as the basis for computing the HPI values. These values exhibited a range from 25.35 (G9) to 2595.68 (G6), with a mean of 263.78 ± 703.51. The elevated HPI values observed in G6 are likely attributable to both natural processes and human activities. Analysis within the broader study area indicated that the estimated HPI values surpassed the critical threshold of 100, as defined by the Ref [55], suggesting that the water is unsuitable for consumption. However, when categorized according to HPI classes, all sampled locations fell within the spectrum ranging from low (HPI < 100) to high (HPI > 100), as illustrated in Fig 4A.

In the investigation of drinking water samples from various stations, the Health Risk Index (HEI) values for eight analyzed metals were evaluated (Fig 4C). Across the entirety of stations surveyed, the HEI values were found to be below the threshold of 10, indicative of low pollution levels, reflective of the relative safety of the majority of the sampled drinking water sources. However, notable exceptions were observed at stations G5 and G6. At station G5, the HEI value ranged between 10 and 20, indicating a state of moderate pollution. This suggests a discernible presence of contaminants surpassing the low pollution threshold, albeit not reaching levels of high risk. Conversely, station G6 exhibited HEI values surpassing 20, indicating a considerably elevated level of pollution, thereby signifying a high-risk scenario concerning water quality.

In Fig 4B, the mean C_deg_ values provide crucial insights into the levels of metal concentration found in tube well waters. The calculated mean C_deg_ value across all samples was determined to be 10.29 ± 38.46. This figure demonstrates a significant level of contamination in the tube well waters sampled. Furthermore, it is worth noting the wide range of C_deg_ values observed among the study’s locations. The range varied from −5.60 in G8 to a strikingly higher value of 137.40 in G6. G6 contains 0.021 mg/L Cd content, which is the highest among all the stations. The possible reason for the highest ERI value in G6 is Cd concentration. This wide range highlights the variability in metal concentration levels among the different groups, with the majority demonstrating zero contamination levels when compared to others.

Expanding upon the ERI values were scrutinized across all surveyed stations, providing a comprehensive understanding of the environmental ramifications associated with metal contamination in the water sources. Overall, the ERI values indicated predominantly lower ecological risks across the sampled stations, indicative of a relatively benign ecological impact, with the exception of station G6 (Fig 4D). At station G6, the RI values exceeded the threshold of 150, signifying a notable escalation in ecological risk and indicating considerable pollution levels within the aquatic ecosystem [12].

### Assessment of health risk indices in water samples

Table 4 presents the results pertaining to the Hazard Quotient via HQ_Oral_, HQ_Dermal_ and Hazard Index (HI) derived from the assessment of various metals across all exposure routes for both adults and children. Notably, the HQ_Oral_ and HQ_Dermal_ values for all metals investigated in both adult cohorts (comprising both males and females) as well as in children were found to be below the safety thresholds (< 1). However, it is pertinent to highlight that the highest HQ_Oral_ value recorded for arsenic (As) among children approached the threshold limit at 7.33E-01, indicating that while the value remains within acceptable limits, children may be more vulnerable to long-term exposure effects due to their physiological sensitivity. Even though the value is technically below the threshold, it is high enough to raise concerns about precautions, especially considering children’s increased susceptibility to toxicants due to their developing physiology. The cumulative and long-term exposure, particularly if consumed on a daily basis, and the non-carcinogenic consequences of arsenic (such as effects on the immune system, neurological system, and development) can be more obvious in younger people. The observed trends suggest that while the majority of the evaluated values remain within the realm of safety, there exists a foreseeable concern regarding the ingestion of certain metals by children [56]. It is imperative to note that in accordance with established protocols [57], an HI value below 1 is generally deemed safe for human consumption. Conversely, an HI exceeding 1 could signify potential hazards to health. In our investigation, the amalgamated risk assessment metric, HI, calculated across all metals under scrutiny, consistently fell below the acceptable threshold (<1) [58], as depicted in Table 4. This outcome implies that the cumulative exposure to the studied metals does not present a significant health risk, aligning with established safety criteria. Consequently, although the collective exposure to the examined metals does not exceed established health risk thresholds, the proximity of arsenic ingestion levels to the safety limit in children indicates a potential concern that warrants further attention and targeted monitoring.

**Table 4:** Estimated hazard quotient (HQ) and hazard index (HI) of heavy metals from drinking water in Bangladesh.

Metals	Male			Female			Child		
	HQ Oral	HQ Dermal	HI	HQ Oral	HQ Dermal	HI	HQ Oral	HQ Dermal	HI
Pb	1.09E-03	7.78E-05	1.17E-03	1.03E-03	1.03E-04	1.14E-03	2.22E-03	7.54E-05	2.29E-03
Cd	6.07E-05	4.32E-05	1.04E-04	5.74E-05	5.74E-05	1.15E-04	1.23E-04	4.19E-05	1.65E-04
Cu	1.21E-05	8.58E-06	2.06E-05	1.14E-05	1.14E-05	2.28E-05	2.45E-05	8.32E-06	3.28E-05
Zn	6.68E-06	2.86E-06	9.54E-06	6.32E-06	3.79E-06	1.01E-05	1.36E-05	2.77E-06	1.63E-05
Mn	4.28E-05	3.05E-05	7.33E-05	4.05E-05	4.05E-05	8.09E-05	8.69E-05	2.96E-05	1.16E-04
Fe	8.54E-02	6.09E-02	1.46E-01	8.08E-02	8.08E-02	1.62E-01	1.74E-01	5.90E-02	2.33E-01
Cr	3.35E-03	1.68E-02	2.02E-02	3.61E-03	1.81E-02	2.17E-02	2.20E-02	3.57E-01	1.79E-01
As	3.66E-03	2.36E-02	4.33E-02	5.04E-02	3.62E-02	4.86E-02	7.33E-01	3.34E-01	4.77E-01

The assessment of carcinogenic risk (CR) within the context of this study delves into the likelihood or probability of individuals developing cancer over their lifetime due to exposure to specific substances or factors known as carcinogens, as elucidated by the Ref [1]. This evaluation entails scrutinizing the intricate relationship between exposure to a given substance or hazard and the subsequent development of cancer [14,59]. Integral to this assessment are considerations of various factors including the dosage and duration of exposure, as well as individual susceptibility predicated upon genetic predisposition, lifestyle choices, and other pertinent variables. Guided by the standards delineated by the Ref [60] United States Environmental Protection Agency (USEPA) in 1989, carcinogenic risk (CR) and total carcinogenic risk (TCR) values are interpreted accordingly. CR and TCR values falling below 10^−6^ are considered negligible, those ranging between 10^−6^ and 10^−4^ are deemed permissible, while values surpassing 10^−4^ are flagged as potentially hazardous to human health [13]. Upon examination, the mean CR values computed for both adult cohorts (comprising both males and females) and children in all three regions were found to be within the permissible limit as per USEPA guidelines (Table 5). This observation underscores a level of reassurance regarding the assessed carcinogenic risks associated with the ingestion of the aforementioned metals via water consumption routes.

**Table 5:** Estimated total cancer risk (CR) of heavy metals from tube well water in Dhaka division, Bangladesh.

Locations	Male		Female		Child	
	Pb	Cd	Pb	Cd	Pb	Cd
Dhamrai	3.76E-09	2.03E-09	3.55E-09	1.92E-09	1.53E-09	8.25E-10
Kaliakair	3.75E-08	2.59E-08	3.54E-08	2.45E-08	1.52E-08	1.05E-08
Savar	1.72E-09	1.72E-09	1.63E-09	1.63E-09	6.99E-10	6.98E-10

### Pollution source identification of studied metals

**Fig 5:**
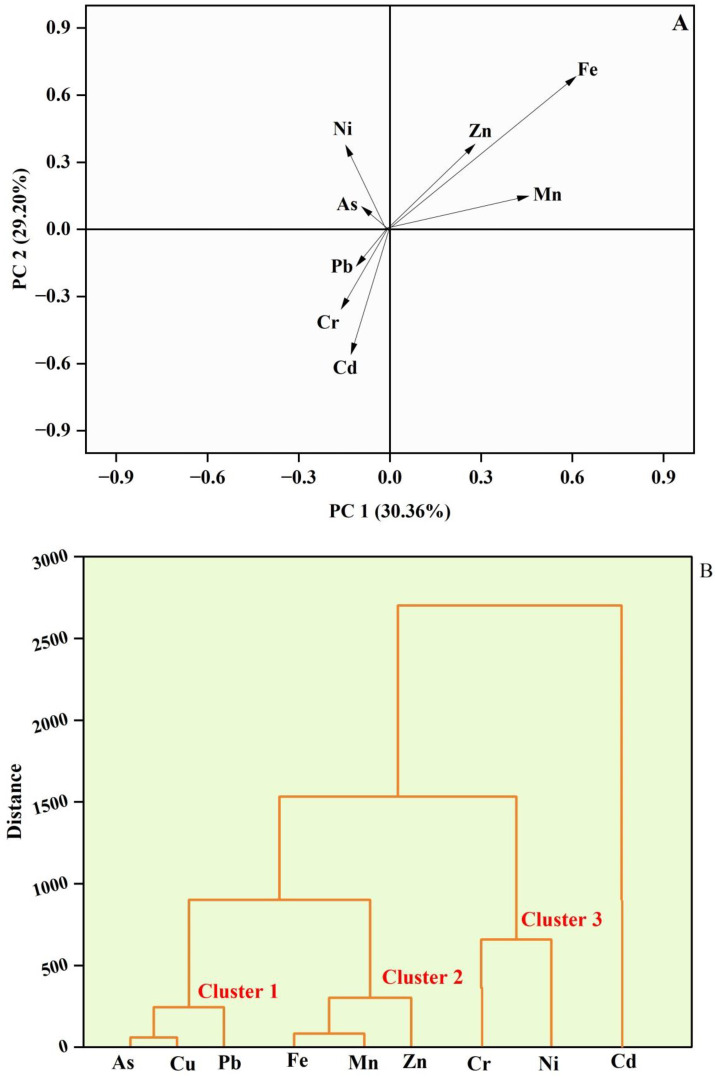
Principal component analysis (PCA) biplot (A) and Cluster analysis (B) of detrimental elements in the drinking water samples of the selected area.

The identification of potential sources of the metals under study was conducted through principal component analysis (PCA) and a Pearson correlation matrix. To evaluate the extent of contamination and identify the origins of the metals, four principal components (PCs) with eigenvalues exceeding 1 were extracted using PCA. In this analysis, Fe, Mn and Zn exhibited notably high positive loadings in the first principal component (PC1), accounting for 30.36% of the total variation (Fig 3A). A strong correlation coefficient of r = 0.97 supported their association, suggesting contributions from both geological formations and nearby industrial activities such as mining, metallurgy, and manufacturing [10,11]. Pb demonstrated a substantial positive loading in the second principal component (PC2), explaining 29.20% of the observed variation (Fig 5A). Industries including mining, smelting, battery manufacturing and metal recycling are recognized as major sources of both zinc and lead [10,61].

The dendrogram in Fig 5B illustrated the hierarchical clustering of identified metals based on their similarity or dissimilarity, represented by the vertical distance on the y-axis. Metals that clustered closely together, such as As, Cu and Pb were likely linked to similar contamination sources, such as industrial discharges, mining activities or the use of pesticides and fertilizers in agriculture [10,11]. Similarly, the grouping of Fe, Mn and Zn suggested they might have originated from natural sources like soil erosion or from anthropogenic activities such as steel manufacturing, battery disposal or sewage effluent [10,12]. The pairing of Cr and Ni indicated potential contamination from industrial processes like metal plating, manufacturing or tannery waste [10,11]. Cd which stood out as a distinct cluster, likely represented contamination from specific sources such as phosphate fertilizers, battery waste, or industrial emissions [11]. Metal plating, battery recycling, tanning, and machinery production are all frequent industrial activities that release Pb, Ni, Cr, Cu, and Zn into the environment via untreated effluents or leaching from waste disposal sites. Sewage and urban runoff, particularly from corroded plumbing, stormwater drainage, and household garbage containing Pb, Zn, and Cu, can increase contamination [62].

## Conclusions

The study conducted an assessment of drinking water (TW) contamination levels in an industrial zone of Bangladesh, considering ecological and human health risks along with corresponding indices. The pH, EC, and TDS levels observed in the water samples all aligned with the drinking water standards set forth by the WHO. Upon comparison of the levels of studied metals in the waters with established standards, it was observed that the mean concentrations of Pb, Fe and Mn in the samples exceeded the recommended limits. Conversely, the average concentrations of As, Cd, Ni, Cu and Zn were lower than those reported in drinking waters from various locations. The evaluation of pollution indices, including the HPI, HEI, C_deg_ and ERI revealed significant contamination at the majority of the surveyed sites. Health risk assessment, conducted through HQ, HI and CR, indicated that most values were within safe limits, except for arsenic (As) carcinogenic risk in children. Both univariate and multivariate analyses highlighted the role of geological formations, as well as contributions from nearby chemical industries and croplands, as sources of metallic pollution. The study’s findings highlight the urgent need for coordinated action from governmental organization and local communities in order to reduce the hazards and protect the area’s natural integrity and public health. In Bangladesh, tube well installation and use are governed by the Ground Water Management Ordinance of 1985 and the Bangladesh Water Act of 2013. These laws emphasize the importance of licensing, correct installation methods, and sustainable water resource preservation while also protecting public health and the environment. The findings of this study emphasize the pressing need for coordinated action involving relevant stakeholders, including governmental agencies, industries, and local communities, to mitigate the identified risks and safeguard both ecological integrity and public health in the studied area. Failure to act decisively could have far-reaching consequences, underscoring the importance of proactive measures to address drinking water contamination in industrial zones.

## Limitations

This study has several limitations that must be addressed when evaluating the findings. First, arsenic speciation was not conducted; only total arsenic and other metal concentrations were measured in water. Second, while the study comprised 12 sites, this sampling design may not have adequately captured the spatial and seasonal diversity of metal exposure in the industrial area of Dhaka. Third, the deterministic approach utilized in health risk assessment excludes variability or uncertainty in exposure parameters that probabilistic models such as Monte Carlo simulation can better capture. Fourth, detailed speciation analysis of As and other heavy metals was not conducted to better understand their bioavailability and potential health risks. Consequently, cumulative exposure from all pathways, though potentially important in more contaminated areas, was not evaluated in this study. These limits are mostly due to logistical and economic constraints. Future study should include metal speciation, probabilistic modeling, expanded spatiotemporal sampling, and site-specific bioaccumulation studies.

## Supporting information

S1 Table Concentrations of Standard Reference Material (SRM NIST 1643f: Trace Elements in Water), and recoveries for metals. (DOCX)

S2 Table Risk indices employed in the current investigation. (DOCX)

S3 Table Heavy metal concentration (mg/L) in studied drinking water samples (TW) from twelve stations. (DOCX)

S4 Table Heavy metal concentration (mg/l) for groundwater’s from deep tube wells in Dhaka city. (DOCX)
