# Supplementary material for: Arsenic and heavy metal contamination in drinking water from an industrial zone in Dhaka District, Bangladesh
Source: PLoS One. 2025 Oct 16;20(10):e0332601. doi: 10.1371/journal.pone.0332601 (PMC12530603; doi:10.1371/journal.pone.0332601)
Supplement: S1 Table — (DOCX) [file pone.0332601.s001.docx]

**S1 Table. Concentrations of Standard Reference Material (SRM NIST 1643f: Trace Elements in Water), and recoveries for metals.**

| **Metal** | **Unit** | **Certified Mass Concentration Value** | **Recovery Range (%)** |
| --- | --- | --- | --- |
| As | (µg/L) | 57.42 ± 0.38 | 97.21-102.21 |
| Al | (µg/L) | 133.8 ± 1.2 | 97.33-101.78 |
| Be | (µg/L) | 13.67 ± 0.12 | 97.67-104.21 |
| Cd | (µg/L) | 5.89 ± 0.13 | 96.21-102.02 |
| Cr | (µg/L) | 18.50 ± 0.10 | 97.87-100.87 |
| Cu | (µg/L) | 21.66 ± 0.71 | 97.87-102.76 |
| Co | (µg/L) | 25.30 ± 0.17 | 98.77-103.08 |
| Fe | (µg/L) | 93.44 ± 0.78 | 95.97-100.38 |
| Pb | (µg/L) | 18.488 ± 0.084 | 97.87-101.95 |
| Mn | (µg/L) | 37.14 ± 0.60 | 96.22-102.05 |
| Ni | (µg/L) | 59.8 ± 1.4 | 97.21-102.78 |
| Se | (µg/L) | 11.700 ± 0.081 | 98.12-101.57 |
| V | (µg/L) | 36.07 ± 0.28 | 97.96-101.09 |
| Zn | (µg/L) | 74.4 ± 1.7 | 95.76-102.56 |
