# Supplementary material for: Arsenic and heavy metal contamination in drinking water from an industrial zone in Dhaka District, Bangladesh
Source: PLoS One. 2025 Oct 16;20(10):e0332601. doi: 10.1371/journal.pone.0332601 (PMC12530603; doi:10.1371/journal.pone.0332601)
Supplement: S2 Table — (DOCX) [file pone.0332601.s002.docx]

**S2 Table. Risk indices employed in the current investigation.**

| **Ecological Risk Indices** | | | | **Health Risk Indices** | | | |
| --- | --- | --- | --- | --- | --- | --- | --- |
|  | **Heavy metal pollution index (HPI)** | **Heavy metal evaluation index (HEI)** | **Degree of contamination index (C_deg_)** | | **Ecological risk index (ERI)** | **Hazard quotient (HQ)and hazard index (HI)** | **Carcinogenic risk (CR)** |
| Objectives | To assess the level of heavy metal pollution in a given area, typically in soil or water [1] | To measure the heavy metal pollution in a given environment [2]: | To measure the contamination based on the concentrations of various pollutants, such as heavy metals or organic compounds, relative to established guidelines or background levels [3]. | | To measure the potential harm to ecosystems resulting from exposure to contaminants. | To provide an indication of whether the level of exposure to a single pollutant exceeds a safe threshold set by health guidelines [4]. | To represent the probability or likelihood that an individual will develop cancer as a result of exposure to a particular carcinogen over a specified period of time [5]. |
| Formula | $HPI=\sum_{i=1}^{n} \frac{Ci}{Ri}$× *Wi*​ [2] | $HEI=\sum_{i=1}^{n} \frac{Mi}{MACi}$ | $C_{d}$=$\sum_{i=1}^{n} Cfi$  $Cfi=\frac{Mi}{MACi}$-1 | | $E_{r}^{i}=T_{r}^{i}\times CF$  $CF=\frac{C_{metal}}{C_{background}}$  $ERI=\sum E_{r}^{i}$ | $HQ=\frac{CDD}{RfD}$  $HI={HQ}_{\mathrm{Ingestion}}+{HQ}_{\mathrm{Inhalation}}+{HQ}_{\mathrm{Dermal}}$ | $CR=CDD\times SF$  $TCR=\sum CR$ |
| Explanation | n = Number of heavy metals,  C_i_ = Concentration of heavy metal in the sample,  R_i_ = Reference concentration or guideline value for heavy metal,  W_i_= Weight assigned to heavy metal. | MAC_i_  is the monitored value and maximum admissible concentration of the ith HM | Cf_i_ is the contamination factor for the ith HM | | Er_i_s the potential ecological risk factor and is the toxic response factor of studied metals. It was determined for Cu=Pb=Ni=Co=5, Mn=Zn=1, As=10, and Cr=2. | RfD reference doses of Pb, Cd, Cr, As, Mn, Fe, Co, Ni, Cu and Zn for ingestion pathway and CDD is the Chronic Daily Dose, representing the estimated amount of the pollutant that an individual might be exposed to on a daily basis over a long period (often a lifetime). | SF is the carcinogenic slope factor set by Ref [6]. |
| Pollution criteria | HPI < 100 represents low pollution.  HPI= 100 is the threshold value at which harmful health consequences are probable.  HPI > 100 indicates the water is unsuitable for consumption. | HEI <10 is low pollution,  HEI= 10-20 for moderate pollution and  HEI >20 is high pollutions. | Cd <8 denotes low contamination,  8≤ Cd≤ 16 denotes moderate contamination,  Cd= 16-32 denotes considerable and  32≥ Cd for high contamination | | Er< 40: Low ecological risk  Er ≤80: moderate ecological risk;  80<Er≤160: appreciable ecological risk;  160 <Er ≤320: high ecological risk; Er>320: serious ecological risk and  RI <150: Low pollution;  150<RI<300: Considerable pollution;  300<RI<600: High pollution;  RI ≥600: Very high pollution [7] | HQ < 1, no adverse health effects; for HQ ≥ 1, there could be a likelihood of possible health hazards.  HI > 10, high risk for its consumers. | Risk levels for carcinogens range from 10^-4^  to 10^-6^. |

**References**

1. Sheykhi V, Moore F. Geochemical characterization of Kor River water quality, fars province, Southwest Iran. Water Quality, Exposure and Health. 2012; 4, 25-38.; 2. Ameh EG. Geo-statistics and heavy metal indexing of surface water around Okaba coal mines, Kogi State, Nigeria. 2013; 3. Backman B, Bodiš D, Lahermo P, Rapant S, Tarvainen T. Application of a groundwater contamination index in Finland and Slovakia. Environmental Geology. 1998; 36, 55-64.; 4. Gad A, Saleh A, Farhat HI, Dawood YH, Abd El Bakey SM. Spatial Distribution, Contamination Levels, and Health Risk Assessment of Potentially Toxic Elements in Household Dust in Cairo City, Egypt. Toxics. 2022; 10(8), 466.; 5. Mugudamani I, Oke SA, Gumede TP. Influence of Urban Informal Settlements on Trace Element Accumulation in Road Dust and Their Possible Health Implications in Ekurhuleni Metropolitan Municipality, South Africa. Toxics. 2022; 10(5), 253.;6. USEPA. Regional Screening Level (RSL) Summery Table. Washington, DC, USA. Available online: https://epa prgs.ornl. gov/chemicals/download/master _sl_table _run _JUN 2011.pdf (accessed on 5 December 2021). 2011.; 7. Hakanson L. An ecological risk index for aquatic pollution control. A sedimentological approach. Water research. 1980; 14(8), 975-1001.
