# Supplementary material for: Arsenic and heavy metal contamination in drinking water from an industrial zone in Dhaka District, Bangladesh
Source: PLoS One. 2025 Oct 16;20(10):e0332601. doi: 10.1371/journal.pone.0332601 (PMC12530603; doi:10.1371/journal.pone.0332601)
Supplement: S3 Table — (DOCX) [file pone.0332601.s003.docx]

**S3 Table. Heavy metal concentration (mg/L) in studied drinking water samples (TW) from twelve stations.**

| **Locations** | **Heavy metal concentration (mg/L)** | | | | | | | | |
| --- | --- | --- | --- | --- | --- | --- | --- | --- | --- |
|  | **As** | **Pb** | **Cd** | **Cr** | **Cu** | **Ni** | **Zn** | **Mn** | **Fe** |
| G1 | 0.001 | 0.047 | 0.0003 | 0.002 | 0.002 | 0.002 | 0.196 | 0.053 | 0.152 |
| G2 | 0.013 | 0.043 | 0.001 | 0.003 | 0.006 | 0.002 | 0.080 | 0.170 | 9.310 |
| G3 | 0.004 | 0.023 | 0.00004 | 0.004 | 0.005 | 0.004 | 0.220 | 0.095 | 8.201 |
| G4 | 0.004 | 0.026 | 0.001 | 0.001 | 0.022 | 0.002 | 0.112 | 0.251 | 8.752 |
| G5 | 0.0002 | 0.019 | 0.0002 | 0.002 | 0.129 | 0.002 | 0.064 | 0.926 | 7.421 |
| G6 | 0.001 | 1.358 | 0.021 | 0.004 | 0.009 | 0.001 | 0.04 | 0.064 | 5.324 |
| G7 | 0.004 | 0.01 | 0.00004 | 0.003 | 0.003 | 0.001 | 0.008 | 0.020 | 10.842 |
| G8 | 0.01 | 0.007 | 0.0002 | 0.002 | 0.002 | 0.002 | 0.013 | 0.094 | 1.251 |
| G9 | 0.001 | 0.008 | 0.001 | 0.002 | 0.002 | 0.001 | 0.005 | 0.068 | 9.126 |
| G10 | 0.001 | 0.017 | 0.0002 | 0.001 | 0.001 | 0.002 | 0.038 | 0.185 | 2.482 |
| G11 | 0.0004 | 0.028 | 0.001 | 0.003 | 0.003 | 0.002 | 0.017 | 0.24 | 2.302 |
| G12 | 0.001 | 0.011 | 0.0002 | 0.002 | 0.012 | 0.003 | 0.023 | 0.271 | 0.590 |
