# Supplementary material for: Arsenic and heavy metal contamination in drinking water from an industrial zone in Dhaka District, Bangladesh
Source: PLoS One. 2025 Oct 16;20(10):e0332601. doi: 10.1371/journal.pone.0332601 (PMC12530603; doi:10.1371/journal.pone.0332601)
Supplement: S4 Table — (DOCX) [file pone.0332601.s004.docx]

**S4 Table. Heavy metal concentration (mg/l) for groundwaters from deep tubewells in Dhaka city.**

| **Locations** | **Heavy metal concentration (mg/L)** | | | | | | | | |  |
| --- | --- | --- | --- | --- | --- | --- | --- | --- | --- | --- |
|  | **As** | **Pb** | **Cd** | **Cr** | **Cu** | **Ni** | **Zn** | **Mn** | **Fe** |  |
| Gazipur | 0.003±0.004 | 0.133±0.370 | 0.002±0.006 | 0.002±0.001 | 0.016±0.034 | 0.002±0.001 | 0.068±0.070 | 0.203±0.233 | 5.479±3.740 | Present study |
| Azimpur | < 0.005 | 0.00028 | 0.00004 | 0.0024 | 0.001 | 0.0021 | 0.02 | 0.027 | 0.021 | [1] |
| Mohammadpur | < 0.005 | 0.00037 | 0.00006 | 0.0007 | < 0.001 | 0.0009 | 0.005 | 0.017 | 0.232 |  |
| BIBM | < 0.005 | 0.00023 | 0.00003 | 0.0086 | 0.002 | 0.0011 | 0.097 | 0.066 | 0.248 |  |
| Banani | < 0.005 | 0.0002 | 0.00003 | 0.0031 | 0.002 | 0.0006 | 0.013 | 0.021 | 0.024 |  |
| Circuit House | < 0.005 | 0.00017 | 0.00002 | 0.0032 | 0.001 | 0.001 | 0.006 | 0.058 | 0.097 |  |

[1]. Kinniburgh DG, Smedley PL (Eds.). Arsenic contamination of groundwater in Bangladesh: Final report (Vol. 2). British Geological Survey. 2001. <https://www.bgs.ac.uk/arsenic/Bangladesh>.
